# Education and training of healthcare staff in the knowledge, attitudes and skills needed to work effectively with breastfeeding women: a systematic review

**DOI:** 10.1186/s13006-016-0097-2

**Published:** 2017-02-02

**Authors:** Anna Gavine, Steve MacGillivray, Mary J. Renfrew, Lindsay Siebelt, Haggi Haggi, Alison McFadden

**Affiliations:** 1 Evidence Synthesis Training and Research Group, School of Nursing and Health Sciences, University of Dundee, Dundee, UK; 2 Mother and Infant Research Unit, School of Nursing and Health Sciences, University of Dundee, Dundee, UK

**Keywords:** Breastfeeding, Healthcare staff, Support, Education, Training, Knowledge, Attitudes

## Abstract

**Background:**

Current evidence suggests that women need effective support to breastfeed, but many healthcare staff lack the necessary knowledge, attitudes and skills. There is therefore a need for breastfeeding education and training for healthcare staff. The primary aim of this review is to determine whether education and training programs for healthcare staff have an effect on their knowledge and attitudes about supporting breastfeeding women. The secondary aim of this review was to identify whether any differences in type of training or discipline of staff mattered.

**Methods:**

A systematic search of the literature was conducted using the Cochrane Pregnancy and Childbirth Group’s trial register. Randomised controlled trials comparing breastfeeding education and training for healthcare staff with no or usual training and education were included if they measured the impact on staff knowledge, attitudes or compliance with the Baby Friendly Hospital Initiative (BFHI).

**Results:**

From the 1192 reports identified, four distinct studies were included. Three studies were two-arm cluster-randomised trials and one was a two-arm individual randomised trial. Of these, three contributed quantitative data from a total of 250 participants. Due to heterogeneity of outcome measures meta-analysis was not possible. Knowledge was included as an outcome in two studies and demonstrated small but significant positive effects. Attitudes towards breastfeeding was included as an outcome in two studies, however, results were inconsistent both in terms of how they were measured and the intervention effects. One study reported a small but significant positive effect on BFHI compliance. Study quality was generally deemed low with the majority of domains being judged as high or unclear risk of bias.

**Conclusions:**

This review identified a lack of good evidence on breastfeeding education and training for healthcare staff. There is therefore a critical need for research to address breastfeeding education and training needs of multidisciplinary healthcare staff in different contexts through large, well-conducted RCTs.

## Background

Breastfeeding is important to save lives and improve the health and wellbeing of women and infants across the globe [1]. However, data suggest that few infants are breastfed to WHO recommendations [2], which include initiation within the first hour after birth, exclusive breastfeeding for the first six months, with continued breastfeeding along with appropriate complementary foods up to two years of age or beyond [1]. Healthcare staff play a critical role in supporting women to breastfeed, but to do this effectively they need appropriate knowledge, such as knowledge of the health outcomes associated with different methods of infant feeding, and the physiological process of lactation, combined with positive, non-judgemental attitudes and effective communication, information provision and practical support skills [3, 4]. However, there is evidence that many health workers lack such knowledge, attitudes and skills [5–10].

Education and training for all disciplines of staff providing breastfeeding support is, therefore, an essential component of breastfeeding support programmes. This includes multi-faceted structured programmes of breastfeeding support, such as the UNICEF/WHO Baby Friendly Hospital initiative (BFHI), which has been shown to be effective in improving breastfeeding outcomes [11–14]. BFHI stipulates that all healthcare staff should be trained to implement best practice breastfeeding policies [15]. To meet BFHI criteria, breastfeeding education and training courses should be of at least 18-h duration with a minimum of three hours supervised clinical practice [15]. Dykes recommends that breastfeeding education programmes for healthcare staff should provide opportunities for critical reflection to facilitate integration of embodied, vicarious, practice-based and theoretical knowledge [16].

However, evidence for the effectiveness of breastfeeding education and training for healthcare staff is lacking. A systematic review of structured compared to non-structured breastfeeding programmes found no randomised controlled trials of the effect of training [11]. A systematic review by Spiby et al. examined the effects of training, education and practice change interventions with health staff and concluded that, due to methodological limitations, there was insufficient evidence to draw conclusions about overall benefit or harm of the interventions [17]. The focus of both of these reviews was the effect of interventions on breastfeeding outcomes rather than on the knowledge, attitudes and skills of participants [11, 17]. While breastfeeding rates are a more outcome, there are many other factors that influence breastfeeding rates. Therefore we chose to focus on changes in knowledge, attitudes and skills to better understand the evidence of the direct effects of education and training. One review reported positive effects of continuing breastfeeding education on the knowledge and skills of nurses and midwives, and on BFHI compliance [18]. However, this review has methodological weaknesses; for example, the outcomes of interest are not clearly reported; two published trials were double-counted (i.e. separate publications of the same trial were counted as individual studies); and significance levels were not reported.

While it is unlikely that education and training of healthcare staff on its own will improve breastfeeding rates, breastfeeding support programmes are unlikely to be effective if the staff delivering them are not equipped with relevant knowledge, attitudes and skills. Therefore it is critical to understand which approaches to breastfeeding education and training are effective in improving multidisciplinary healthcare staff’s knowledge, attitudes and skills as this acts as a proxy measure of programme effectiveness.

The primary aim of this review of randomised controlled trials is to determine whether education and training programs for healthcare staff has an effect on their knowledge and attitudes about supporting breastfeeding women compared to no or usual training (comparator). The secondary aim of this review was to identify if there were differences in terms of the type of training and the discipline of the healthcare staff.

## Methods

The methods for this systematic review follow the guidance detailed by the Cochrane Collaboration [19].

### Eligibility criteria

In order to be included in this review studies had to examine the impact that training of healthcare staff, who come into contact with mothers and infants, about breastfeeding and supportive feeding practices had on the following primary outcome measures: 1) breastfeeding knowledge or 2) attitudes towards breastfeeding. Secondary outcomes were: 1) practice of BFHI steps 3–9 (see Table 1) or 2) adherence to the provisions of the International Code of Marketing of Breastmilk Substitutes [20]. Studies had to be either individual- or cluster-randomised controlled trials which compared training for healthcare staff with no or usual training.

**Table 1:** Ten steps to successful breastfeeding

Every facility providing maternity services and care for newborn infants should:
1. Have a written breastfeeding policy that is routinely communicated to all health care staff.
2. Train all health care staff in skills necessary to implement this policy.
3. Inform all pregnant women about the benefits and management of breastfeeding.
4. Help mothers initiate breastfeeding within half an hour of birth.
5. Show mothers how to breastfeed, and how to maintain lactation even if they should be separated from their infants.
6. Give newborn infants no food or drink other than breast milk, unless medically indicated.
7. Practise rooming-in - that is, allow mothers and infants to remain together - 24 h a day.
8. Encourage breastfeeding on demand.
9. Give no artificial teats or pacifiers (also called dummies or soothers) to breastfeeding infants.
10. Foster the establishment of breastfeeding support groups and refer mothers to them on discharge from the hospital or clinic.

Source**:** *Protecting, Promoting and Supporting Breastfeeding: The Special Role of Maternity Services,* a joint WHO/UNICEF statement published by the World Health Organization [38]

Studies which involved training for healthcare staff but did not measure the effect on outcomes related to healthcare staff’s knowledge or attitudes or either of the secondary outcomes were considered ineligible. Studies involving other groups that provide breastfeeding education and support (i.e. students, peer/lay workers) were also excluded. No restrictions were placed on language or date of publication.

### Search strategy

We searched the Cochrane Pregnancy and Childbirth Group’s Trials Register by contacting their information specialist who provided a topic specific search for all Cochrane reviews on the subject of breastfeeding. The Cochrane Pregnancy and Childbirth Group’s Trials Register contains trials identified by their information specialist from the following sources: CENTRAL (monthly searches), MEDLINE (Ovid; weekly searches), EMBASE (Ovid; weekly searches), CINAHL (EBSCO; monthly searches), hand searches of 30 journals and proceedings of major conferences, and weekly current awareness alerts for a further 44 journals plus monthly BioMed Central email alerts. The information specialist then assigns each record a topic number based on the study intervention. Searching for the topic number then provides a specific search for each review topic. Full details on the Trials Register are available from the Cochrane Pregnancy and Childbirth Group’s page within the Cochrane library (http://pregnancy.cochrane.org/pregnancy-and-childbirth-groups-trials-register). The list for this review was provided on July 20^th^ 2016.

### Study selection

All records from the lactation/breastfeeding specific search of the Cochrane Pregnancy and Childbirth Group’s Trial Register were imported into Endnote X7 (Thompson Reuters, USA). Two authors independently screened all titles and abstract and any disagreements were then resolved by a third author. Full texts of studies which potentially met the inclusion criteria or where the abstract did not provide sufficient information were retrieved and then independently screened by two reviewers. Again, any disagreements were resolved by a third author. The reference lists of included studies were then examined for any additional studies meeting the inclusion criteria.

### Data extraction

One author extracted data using the Cochrane Pregnancy and Childbirth Group’s data extraction form. A second author checked the completed form and the data were then entered into Revman [21]. Study authors were contacted for any missing information. Data were extracted on sample (including details on setting such as staff group, healthcare facility type, geographical location), intervention, comparison and outcomes measured (including details of scales where available). We extracted data on the following outcomes: 1) healthcare staff’s knowledge of breastfeeding, 2) healthcare staff’s attitudes towards breastfeeding and 3) practice of BFHI steps 3–9. None of the included studies measured adherence to the provisions of the International Code of Marketing of Breastmilk Substitutes.

### Risk of bias assessment

The Cochrane risk of bias tool was used to assess risk of bias in the included studies [22]. This tool assesses the risk of bias across five domains: 1) random sequence generation, 2) allocation concealment, 3a) blinding of participants and personnel, 3b) blinding of outcome assessment, 4) incomplete outcome data, 5) selective reporting and 6) other bias (bias due to problems not covered by 1–5). Each domain was judged at ‘high’, ‘low’ or ‘unclear’ risk of bias. Risk of bias assessment was first conducted by one author (AG) and this was then checked by a second author (AMF). Had a meta-analysis been possible (see below), we would have explored the impact of bias through a sensitivity analysis.

### Statistical analysis

Had studies been sufficiently similar in terms of intervention and outcomes we would have combined the studies in a meta-analysis. However, due to the small number of studies and heterogeneity in the outcome measurement, it was not possible to do a meta-analysis. When data were available the mean difference for outcomes from individual studies was calculated and a Z test for overall effect was performed.

## Results

**Fig. 1:**
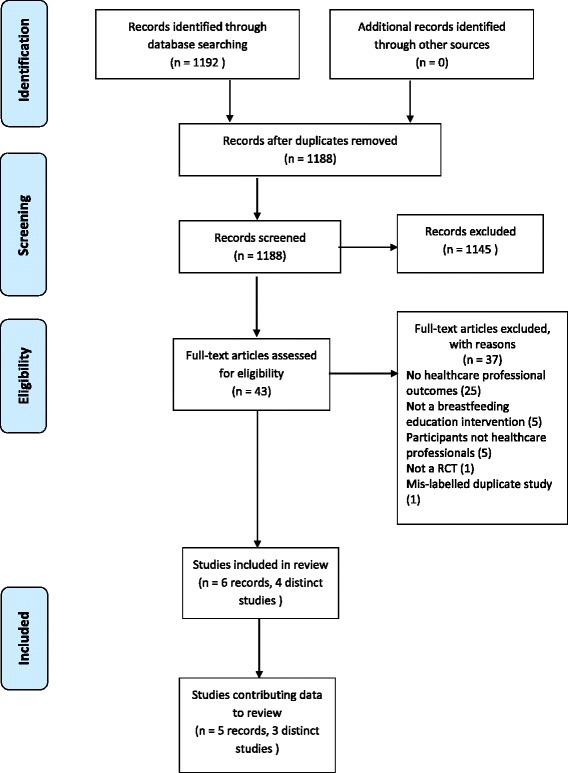
Study Selection Process

The search yielded 1192 reports of which 1188 were unique citations. Of these, 1145 were excluded based on title and/or abstract because they were irrelevant to the review, were interventions that were not focused on breastfeeding education and training for healthcare staff, did not measure healthcare staff outcomes or were not a randomised or cluster-randomised trial. See Fig. 1 for details of the study selection process.

Forty-three full-text articles were reviewed, of which 37 were excluded. The majority of studies (*n* = 25) were excluded as they did not measure healthcare staff’s knowledge, attitudes or practice of BFHI steps 3–9 or adherence to the provisions of the International Code of Marketing of Breastmilk Substitutes. Five studies were excluded because they were not breastfeeding education and/or training interventions. A further five studies were excluded as the participants were not healthcare staff (i.e. peer/lay workers or students). One study was not a RCT and the final study was a duplicate of an included study that had been mislabelled. The search of the reference lists of included studies did not yield any additional studies.

Six separate records for four distinct studies met the inclusion criteria for this review [23–28]. Specifically, there were two records for one study by Ekstrom et al. [23, 27] and two records for one study by Westphal et al. [26] and Taddei et al. [28]. Ekstrom et al. [23] and Westphal et al. [26] were considered the primary references for these studies and the studies will therefore be referred to using these references. Of the four included studies, only three contributed quantitative data in the form of results to our review [23–25]. Westphal et al. did not provide standard deviations or any data that could be used to impute standard deviations so therefore could not contribute data that could be analysed in a meta-analysis (if that had been possible) or for the Z test for overall effect [26].

### Study characteristics

The four studies contained a total of 263 participants [23–26]. As Westphal et al. [26] did not contribute data to the review, there was a total 250 participants from the three studies contributing quantitative data [23–25]. The primary records for each of the included studies were published in English between 1995 and 2008. However a follow-up paper for the study by Ekstrom et al. was published in 2015 [27]. The main characteristics of the four included studies are detailed in Table 2.

**Table 2:** Characteristics and outcomes of included studies

Author and Year	Study design	Setting	Participants	Intervention	Outcomes
				Comparison	
Ekstrom 2005 [23] and Ekstrom 2015 [27]	2-arm cluster-randomised	10 municipalities in South-Western Sweden	Midwives (*n* = 28) and postnatal nurses (*n* = 53).	Process-orientated training programme. Consisted of seven sessions, including discussions on counselling skills and reflection on personal breastfeeding experiences. Participants were encouraged to develop a common breastfeeding policy between the antenatal clinic and the receiving child-health centres. *N* = 36.	Breastfeeding attitudes at 1 year post training through 4 sub-scales: regulating, facilitating, disempowering and breastfeeding antipathy.
				Usual approaches to training and care. No further detail provided. *N* = 45	
Kronborg 2008 [24]	2-arm cluster-randomised	22 municipalities in Western Denmark	Health visitors (*n* = 52)	18 h training ‘Breastfeeding Promotion and Support in a Baby-friendly Hospital’ course by the WHO. Included oral presentations, video presentations, exercises and role play. *N* = 52	Breastfeeding knowledge (including management of breastfeeding practices), indirect measures of attitudes (self-efficacy, subjective norms, behavioural intent and evaluation of importance) and BFHI stage 5 all measured immediately after programme.
				Usual practice and then received the programme at the end of the study. *N* = 57.	
Rea 1999 [25]	2-arm individual randomised trial	Maternity hospital in a low income metropolitan area of Sao Paulo, Brazil.	Healthcare professionals working in maternity services (*n* = 60)	WHO/UNICEF 40 h breastfeeding counselling training course. Delivered as 33 sessions over 40 h and included theoretical and clinical aspects of breastfeeding and training on counselling skills. *N* = 20	Breastfeeding knowledge by a multiple choice test^a^ immediately after the intervention. Observation of clinical and counselling^a^ skills specific to breastfeeding.
				Usual training/care provision – no other details specified. *N* = 40	
Westphal 1995 [26] and Taddei [28]	2-arm cluster-controlled trial	8 hospitals in a metropolitan area of wider Sao Paulo, Brazil.	Healthcare professionals (obstetricians, paediatricians, nurses) in maternity hospitals (*n* = 12)	3 week programme delivered by Santos Lactation Centre based on the Wellstart^TM^ San Diego programme. Covered theoretical and practical aspects of breastfeeding. 66% of time was lectures and remainder was practical sessions. *N* = 4 (cluster level).	Breastfeeding knowledge post-intervention^a^. Compliance of the hospital with the BFHI ten steps measured by structured observations by a researcher and a questionnaire based on staff and mother’s reports in the institutional questionnaire^a^
				No detail provided on comparator. *N* = 4 (cluster level).	

^a^Does not contribute data

1. The studies comprised three two-arm cluster-randomised trials [23, 24, 26] and one two-arm individual-randomised study [25].

#### Participants and setting

Two studies were conducted in the wider metropolitan area of Sao Paulo, Brazil [25, 26], one study was conducted in 13 municipalities in Western Denmark [24], and one study was conducted in 22 municipalities in South-West Sweden [23]. One study specifically focused on midwives and postnatal nurses [23], one study specifically focused on health visitors [24], one study included physicians (paediatricians and obstetricians) and nurses [26] and the final study did not provide details of the healthcare staff receiving the intervention [25]. Three of the studies provided data at the level of individual participants [23–25] giving a total of 250 participants and one study provided data at the level of the cluster with a total of eight clusters [26].

#### Intervention

The interventions generally comprised a combination of lectures, discussion and practical exercises. However, duration ranged considerably from 18 h [24] to 133 h [26]. The content of the intervention varied, with two studies using WHO breastfeeding courses, one the 18-h ‘Breastfeeding Promotion and Support in a Baby-friendly Hospital’ course [24] and the other the WHO 40-h Breastfeeding Counselling course [25]. One study developed its own process-orientated training programme, which included the development of a common breastfeeding policy between the antenatal clinic and the receiving child-health centres [23]. Finally, one study developed a course based on the Wellstart^TM^ Lactation programme which was adapted for use within a Brazilian setting [26]. No details were provided on the characteristics of the person providing the intervention in any of the studies.

### Risk of bias

Studies were generally judged to be at high or unclear risk of bias across the domains. Only one study was judged at low risk of selection bias for random sequence generation and allocation concealment [24]. None of the other three studies provided any information about the random sequence generation or allocation concealment so all studies were judged to be at unclear risk of selection bias [23, 25, 26]. Due to the nature of the study, it was not possible to blind study participants so all four studies were judged to be at high risk of performance bias. Similarly, as three of the studies involved self-report outcomes, detection bias was judged to be at high risk [23–25]. The studies which included data for outcomes collected by researchers did not provide details regarding blinding of outcome assessors [25, 26]. Three studies were judged at low risk of attrition bias, however, one study had a loss to follow-up of over 30% and was therefore judged to be at high risk of bias [23]. None of the included studies had pre-defined outcomes published in a protocol and as such were judged to be an unclear risk of bias. However, one study did not report all the outcomes for each of the time points detailed in the methods section and was therefore judged to be at high risk of bias for selective outcome reporting [23]. There was also no evidence that clustering had been accounted for in the analysis.

### Outcomes and effects of interventions

Outcome assessment varied across the studies both in terms of measurements used and data collection time points, with two studies only conducting some of the follow-up assessments in the groups receiving the interventions [23, 25]. Outcomes measured included breastfeeding knowledge, attitudes towards breastfeeding and compliance with BFHI. Adherence to the provisions of the International Code of Marketing of Breastmilk Substitutes was not included as an outcome in any of the studies. No studies reported the use of a validated instrument.

#### Knowledge

Three studies, using different scales, measured breastfeeding knowledge at baseline and immediately after the intervention [24–26].

Kronborg et al. used a version of the Breastfeeding Knowledge Questionnaire, modified to conditions in Denmark [24]. This included an 11 point scale measuring breastfeeding practices and then three short case studies which measured breastfeeding management. This was conducted at baseline and immediately after the programme [24].

Rea et al. used a 13-item multiple choice questionnaire which was completed before and immediately after the course [25]. Only the intervention group completed it again three months later. Standard deviations were not provided for this outcome and there was insufficient information provided in the paper to impute standard deviations, therefore this outcome does not contribute data to the review. Rea et al. also included an assessment of clinical and counselling skills in a breastfeeding consultation, which can be considered to be an indirect measure of knowledge [25]. This first involved observation of clinical sessions by two researchers who measured and awarded points for the following skills in clinical consultations: clinical history (10 points); assessment of a breastfeed (14 points); non-verbal communication (25 points); listening and learning (25 points); and building confidence and giving support (45 points). Means and standard deviations were available for this outcome, which therefore contributes data to the review. Secondly, 16 items related to observed counselling sessions were also presented, however, no standard deviations were reported and again due to lack of information it was not possible to impute values so this outcome did not contribute data to the review.

Finally, Westphal et al. measured breastfeeding knowledge pre- and post-intervention with a test (no details provided), however, this was only completed by staff receiving the intervention and not the control group, so will not contribute data to the review [26].

##### Effects of the intervention

Two studies contributed data for this outcome, however, as one study utilised a direct measure of knowledge [24] and the other study used what was considered an indirect measure of knowledge [25], it was not considered appropriate to combine the studies in a meta-analysis.

In terms of the direct measure of knowledge, Kronborg et al. reported that health visitors receiving the 18 h WHO ‘Breastfeeding Promotion and Support in a Baby-friendly Hospital’ course had significantly higher* scores post-intervention for both the knowledge questionnaire (Mean difference [MD] = 1.61, 95% CI = 1.03, 2.19, *Z* = 5.46, *p* < 0.00001) and management of breastfeeding practice case studies (MD = 0.51, 95% CI = 0.26, 0.76, *Z* = 4.06, *p* < 0.0001), than staff who did not receive additional training [24].

In relation to the indirect measure of knowledge, Rea et al. reported that healthcare staff receiving the 40 h WHO/UNICEF breastfeeding counselling course had significantly higher scores, which indicate higher levels of knowledge, post-intervention than staff who did not receive any training for all five measures of clinical skills [25]: clinical history taking (MD = 1.47, 95% CI = 0.73, 2.21, *Z* = 3.92, p < 0.0001); assessment of a breastfeed (MD = 1.31, 95% CI = 0.35, 2.27, *Z* = 2.69, *p* = 0.007); non-verbal communication (MD = 1.31, 95% CI = 0.35, 2.27, *Z* = 2.69, *p* = 0.007); listening and learning MD = 7.17, 95% CI = 5.25, 9.09, *Z* = 7.31, *p* < 0.00001; and building confidence and giving support (MD = 11.67, 95% CI = 8.86, 14.48, *Z* = 8.13, p < 0.00001).

#### Attitudes

Two studies measured breastfeeding attitudes and again there was variation in how this was measured. Ekstrom et al. developed a 35-item breastfeeding attitude instrument using four-point Likert scales which consisted of four sub-scales [28]: regulating (participants’ orientation on regulating the mothers’ breastfeeding management); facilitating (making it possible for mothers to manage their own breastfeeding); disempowering (giving advice that disregarded the needs of the mother receiving counselling); and breastfeeding-antipathy (insufficient, basic breastfeeding knowledge and hostile reactions to breastfeeding). Data were collected at baseline and one year after the training had ended in both the intervention and control group, and also immediately after the intervention in the intervention group only.

Kronborg et al. measured the following psychosocial variables which could be argued to be an indirect measure of attitudes towards breastfeeding [24]: self-efficacy; intention to engage in breastfeeding support; subjective norms; and evaluation of the level of importance that the healthcare staff placed on whether or not the mothers who they provided care for breastfeed All of these were measured with a one-item five-point Likert scale, with the exception of self-efficacy which was measured by a five-item five-point Likert scale. All items were measured at baseline and immediately after the intervention. Only questions related to importance of breastfeeding and self-efficacy were measured at the six month follow-up.

##### Effects of the intervention

Two studies contributed data for this outcome, however, as one study used a direct measure of attitudes reported as four separate sub-scales [23] and another used what were considered indirect measures of attitudes [24], the data were too heterogeneous to combine.

In terms of the direct measure of attitudes, Ekstrom et al. reported that at one year post-intervention midwives and postnatal nurses receiving the process-orientated training had significantly improved scores in the facilitating (MD = 0.21, 95% CI = −0.02, 040, *Z* = 2.16, *p* = 0.03) and significantly improved scores in the disempowering (MD = −0.26, 95% CI = −0.51, −0.01, *Z* = 2.05, *p* = 0.04) sub-scales than midwives and postnatal nurses who did not receive additional training [23]. However, no significant differences between the groups were reported for either the regulating (MD = −0.18, 95% CI = −0.40, 0.04, *Z* = 1.62, *p* = 0.10) or breastfeeding antipathy (MD = −0.03, 95% CI = −0.15, 0.09, *Z* = 0.48, *p* = 0.63) sub-scales.

For the indirect measure of attitudes, Kronborg et al. reported that immediately after the intervention, health visitors receiving the 18 h WHO ‘Breastfeeding Promotion and Support in a Baby-friendly Hospital’ course had significantly improved scores for subjective norms supporting breastfeeding (MD = 0.14, 95% CI = 0.02, 0.26, *Z* = 2.28, *p* = 0.02) and evaluation of the importance of breastfeeding (MD = 0.36, 95% CI = 0.06, 0.66, *Z* = 2.36, *p* = 0.02) than health visitors not receiving additional training. However, there were no significant differences in self-efficacy between groups (MD = 0.18, 95% CI = −0.12, 0.48, *Z* = 1.18, *p* = 0.24) [24].

#### Compliance with BFHI steps

Two studies measured outcomes related to BFHI steps. First, Westphal et al. assessed compliance with steps 1–10 through analysis of structured observations by a researcher and through analysis of an institutional questionnaire which includes the following sources of information [26]: interviews with staff in the health team who had managerial positions or direct contact with mothers and newborns; interviews with women who had just given birth; observation of maternity areas; interviews with women who have just given birth;; and interviews with pregnant women at the antenatal clinic. Data were presented at the level of each clinic and not grouped together by intervention and control clinics. Moreover, standard deviations or any information that could be used to impute standard deviations were not reported, so this study does not contribute any data to the review. Secondly, Kronberg et al. measured BFHI step 5 (demonstrating breastfeeding) by asking mothers if they had been given a demonstration of breastfeeding. The outcome was reported as the average value per health visitor (range is 0–1) [24].

##### Effects of the intervention

The one study that contributed data to this outcome reported that health visitors who received the 18 h WHO ‘Breastfeeding Promotion and Support in a Baby-friendly Hospital’ course were significantly more likely to perform a demonstration of how to breastfeed to mothers (MD = 0.28, 95% CI = 0.23, 0.33, *Z* = 10.10, p < 0.00001) than health visitors who did not receive the intervention [24].

## Discussion

This systematic review identified a lack of studies examining the impact of training and education on breastfeeding and supporting feeding practices for healthcare staff. This is in stark contrast to a large number of RCTs which have examined the impact of interventions targeted at mothers that aim to promote initiation (*n* = 23 included trials) and provide support for breastfeeding (*n* = 73 included trials) [13, 29, 30]. This low number may be in part due to restriction of this review to only include randomised trials, whereas previous reviews included further interventions which were evaluated with non-randomised designs [17, 18]. Studies that have used non-randomised designs have reported positive effects. For example, the American Academy of Paediatrics Breastfeeding Residency curriculum reported significant improvements in breastfeeding knowledge, practices and confidence [31]. However, this study is potentially at high risk of bias due to a high rate of attrition and non-reporting of baseline characteristics by group allocation. Similarly, an uncontrolled before-and-after study evaluating the UNICEF/WHO 20-h course for maternity staff reported significant improvements in BFHI steps 4,7 and 8 [32]. However, the lack of a randomized control group gives rise to concerns about selection bias and it is therefore difficult to draw conclusions from either of these studies [31, 32]. Nevertheless, it should be noted that despite the use of randomised designs, the four studies included in this review were judged at unclear or high risk of bias across the majority of the domains [23–26]. Therefore, whilst there were some small but significant positive effects in terms of measures of breastfeeding knowledge [24, 25], four of the seven measures of attitudes towards breastfeeding [23, 24], and performance of BFHI step five [25], this evidence is extremely limited in terms of quality and the small number of studies on which it is based. This therefore represents a major gap in knowledge about effective ways to improve a critically important public health behaviour and ultimately means that women and babies may not receive the help they need.

The lack of evidence related to breastfeeding education of healthcare staff is especially worrying, given that the 2016 *The Lancet* Series on Breastfeeding has recently drawn renewed international attention to the potential of breastfeeding to save lives and prevent disease, and to widespread sub-optimal infant feeding [1]. Recent evidence has also pointed to the positive impact of the BFHI with its emphasis on supportive practice including help with positioning and attachment of the baby at the breast to avoid pain, and maximising milk production through frequent and unrestricted feeding. It is therefore critical that healthcare staff including the whole multidisciplinary team are equipped with these skills [11–14]. While there is evidence that training and education of health staff can improve breastfeeding rates [13, 14, 33], our review shows that there is an urgent need for high quality research to inform the design and delivery of effective education and training opportunities.

In contrast to previous systematic reviews that have focussed on the effects of training and education of healthcare staff on breastfeeding rates [11, 13, 14, 17], our review‘s primary focus was changes in knowledge attitudes and skills. This is important because there is a need to understand how best to teach the complex mix of knowledge, attitudes and skills in a topic area that is often seen as contentious and where healthcare staff may have limited education, diverse views and experiences, and possibly ambivalent feelings. As Dykes discusses, attitudes towards breastfeeding are rooted in personal and vicarious (observation and influence of others) experiences [16]. Particularly in settings where there have been historically low breastfeeding rates, healthcare staff may have had difficult personal experiences of breastfeeding that will have a direct influence on their attitudes. For example, in one study only 45% of doctors and 65% of nurses were convinced that infants should be exclusively breastfed for the first six months [34]. In this context, the lack of high quality evidence to inform pedagogical approaches is of great concern. Furthermore our review found only two trials that measured changes in attitudes as a result of breastfeeding training.

Ambivalent attitudes towards breastfeeding are also influenced by pervasive marketing and increasing global sales of infant formula [13, 35, 36]. The International Code of Marketing of Breastmilk Substitutes and its subsequent resolutions are fundamental to protecting the public and healthcare staff from inappropriate marketing by infant formula companies [20]. It is therefore critical that healthcare staff who are in contact with breastfeeding mothers and babies, their families, and communities understand the International Code of Marketing of Breastmilk Substitutes and their role in its implementation. Without such knowledge, healthcare staff are vulnerable to direct and indirect marketing [13]. However our review found no studies that addressed adherence to the provisions of International Code of Marketing of Breastmilk Substitutes.

This main strength of this review is that it was conducted following Cochrane Collaboration rigorous standards and included only randomised control trials. Limitations include that all of the included studies had significant methodological weaknesses and, due to differences in study interventions and outcome measures, we were unable to conduct a meta-analysis. Nevertheless, this review indicates that we know almost nothing about what forms of education/training would be best, despite hugely varying provision globally. Further research is needed for example to compare different lengths, content, modes of delivery and inclusion of theory and clinical skills. Although not included in this review, it would be relevant to evaluate undergraduate education for example using BFI UK education standards [37]. Education/training interventions need to be underpinned by pedagogical theory relating to areas such as knowledge, attitudes, skills, and countering of negative/contentious public discourse.

## Conclusions

This review identified a lack of good quality evidence to determine whether breastfeeding training and education for healthcare staff can help improve breastfeeding knowledge and attitudes, compliance with BFHI or adherence to the provisions of the International Code of Marketing of Breastmilk Substitutes. Therefore, whilst there were some small but significant positive effects in terms of measures of breastfeeding knowledge, some measures of attitudes towards breastfeeding, and performance of BFHI step five (demonstration of breastfeeding for all women) this evidence is extremely limited in terms of methodological quality and the small number of small studies on which it is based. The lack of evidence in this field represents a major gap in knowledge about a critically important public health behaviour and ultimately means that women and babies are unlikely to receive the help they need consistently.

## Abbreviations

BFHI: Baby friendly hospital initiative
MD: Mean difference
